# Perceptions of Ethical Climate and Research Pressures in Different Faculties of a University: Cross-Sectional Study at the University of Split, Croatia

**DOI:** 10.1007/s11948-017-9987-y

**Published:** 2017-10-25

**Authors:** Mario Malički, Vedran Katavić, Domagoj Marković, Matko Marušić, Ana Marušić

**Affiliations:** 1 Department of Research in Biomedicine and Health, University of Split School of Medicine, Šoltanska 2, 21000 Split, Croatia; 2 Cochrane Croatia, University of Split School of Medicine, Split, Croatia; 3 Department of Anatomy, University of Zagreb School of Medicine, Zagreb, Croatia; 4 Department of Cardiology, University of Split Hospital Centre, Split, Croatia

**Keywords:** Ethical climate, Research integrity, University, Medicine, Engineering, Humanities

## Abstract

We determined the prevailing ethical climate at three different schools of a single university, in order to explore possible differences in the ethical climate related to different research fields: the School of Electrical Engineering, Mechanical Engineering, and Naval Architecture; the School of Humanities and Social Sciences; and the School of Medicine. We used the Ethical Climate Questionnaire to survey the staff (teachers and administration) at the three schools, and used the research integrity and organizational climate (RIOC) survey for early-stage researchers at the three schools. The dominant ethical climate type perceived collectively at the three university schools (response rate 49%, n = 294) was *Laws and professional codes*, which is associated with the cosmopolitan level of analysis and the ethical construct of principle. Individually, the same climate predominated at the schools for engineering and humanities, but the School of Medicine had the *Self*-*interest* ethical climate, which is associated with the individual level of analysis and the egoism ethical construct. In the RIOC survey (response rate 85%; n = 70), early-stage researchers from the three university schools did not differ in their perceptions of the organizational research integrity climate, or in their perceived individual, group or organizational pressures. Our study is the first, to the best of our knowledge, to show differences in perceived ethical climate at a medical school compared to other schools at a university. Further studies are needed to explore the reasons for these differences and how they translate to organizational outcomes, such as job satisfaction, commitment to the institution and dysfunctional behaviour, including research misconduct.

## Introduction

Research integrity and its negative counterpart—research misconduct—are often perceived as the result of an individual(‘s) action(s) (Fanelli 2009). Reports in both research publications and public media outlets most often focus on individual cases (Katavic 2014), without establishing the full context of expected behaviours as well as the responsible conduct of research at all levels: individual, institutional and social (Meriste et al. 2016). While often incredible in hindsight, the feats of all of the perpetrators of scientific misconduct have happened under the (watchful) eyes of their subordinates, peers and superiors (Katavic 2014; Rudolph et al. 2013; Marcus and Oransky 2016) circumventing established (un)written rules. Research misconduct and questionable research practices may be a special problem in small and emerging scientific communities, such as in low- and middle-income countries (Marušić et al. 2011; Ana et al. 2013; Okonta and Rossouw 2014) as the incidence of misconduct may be similar to or higher than that in the high-income countries (Okonta and Rossouw 2014; Ana et al. 2013), but the level of receptiveness to the problem is lower (Magnus et al. 2002).

At the institutional level, it is expected that “institutions should create and sustain environments that encourage integrity through education, clear policies, and reasonable standards for advancement, while fostering work environments that support research integrity” (The Singapore statement on Research Integrity 2010). Research institutions are expected to “promote awareness and ensure a prevailing culture of research integrity” (ALLEA 2017). However, it is not clear how institutional research integrity could be defined (Meriste et al. 2016) or how to best measure it, as the definitions of research ethics, research integrity as well as research misconduct and responsible conduct or research are still debated and are in varied use (Horbach and Halffman 2016; Komic et al. 2015).

Starting from the position that the nature of the immediate research environment may influence one’s (misconduct) behaviour, and urged by the fact that so little was known about institutional ethics, Gaddis et al. (Gaddis et al. 2003) developed two measures of research (ethical) organizational climate for scientific organizations: (1) a measure of the ethical and creative aspects of the organizational research climate, and (2) situational measures influencing the research climate at individual, group and organizational levels, from the point of view of early stage researchers. The elements of the organizational climate were later shown to predict ethics decision making by doctoral students (Mumford et al. 2007), as many young scientists have reported unethical pressures from their work-peers (Nilstun et al. 2010; Hofmann et al. 2013) and their superiors (Kwok 2005; Macfarlane 2017). More recently, Wells et al. (2014) developed a new instrument for assessing the organizational research climate, which includes ethical leadership and framework for research integrity. The instrument was validated in three research-intensive, doctoral-granting universities by surveying over 11 thousand respondents (Wells et al. 2014). It was also successfully used in the research services of healthcare institutions (Martinson et al. 2016). The same instrument was used in the study that found more positive perceptions of research climate by researchers were associated with more positive reports of their research practices (Crain et al. 2013). Most of the studies of organizational research climate involved researchers, but not other professionals who may contribute to (research) integrity climate, such as the administrative staff.

While these newly developed instruments have not been fully validated in different environments and institutions, the oldest and best-validated instrument measuring organizational ethical climate (Victor and Cullen 1987; Cullen et al. 1993) had rarely been tested in academic and research institutions. The ethical climate questionnaire was developed in 1987 (Victor and Cullen 1987), and expanded in 1993 (Cullen et al. 1993) to measure a type of an organizational climate that encapsulates shared perceptions of ethically correct behaviours and ways by which ethical issues should be handled within an organization (Victor and Cullen 1987). Ethical climate theory (ECT) is based on a model that incorporates two theoretical dimensions, that of ethical philosophy, with the criteria of *egoism*, *benevolence* and *principle*, and the sociological theory dimensions of three loci: *individual*, *local* and *cosmopolitan* (Simha and Cullen 2012). Ethical climate has been shown to influence a variety of work-related attitudes and behaviours, such as job satisfaction, organizational commitment, and ethical behaviour (reviewed in Simha and Cullen 2012), and the theoretical tenets of the ECT have been confirmed in a meta-analysis of consequences of perceived ethical climates (Martin and Cullen 2006).

Although ethical climate has been measured in a variety of professional settings, mostly business (reviewed in Simha and Cullen 2012) and some in healthcare settings (Atabay et al. 2015; Abou Hashish 2017; Dinc and Huric 2016; Koskenvuori et al. 2017), very few studies have looked at ethical climates of higher education academic/educational institutions (Acharya 2005; Al Omari 2013; Acar et al. 2016). These studies did not specify the university departments surveyed (Al Omari 2013; Acar et al. 2016), and only the study of Acharya et al. specifically surveyed professors and students in a dental school.

The aim of our study was to determine the prevailing ethical climate at three different schools of a single university, in order to explore possible differences in ethical climate related to different research fields predominating at the three schools: engineering, humanities, and medicine. We were particularly interested in the evaluation of the ethical climate in a medical school, where ethics is formally taught in the curriculum (Grković et al. 2012) and is an important part of the deontology of the medical profession (Davey 2001). We also explored another measure of climate specific for research and academic organizations: organizational climate and research integrity pressures as perceived by early-stage researchers, as a sensitive indicator of the research ethics environment at their workplace (Gaddis et al. 2003).

## Methods

### Participants

The participants in the ethics climate study were the faculty and administration personnel from three Schools of the University of Split in Split, Croatia. The participants in the study of the organizational climate and pressures for research were junior (doctoral and early postdoctoral) researchers from these Schools.

The ethics approval for the study was obtained from the University of Split School of Medicine. The management of three Schools from the University of Split, the School of Electrical Engineering, Mechanical Engineering, and Naval Architecture; the School of Humanities and Social Sciences; and the School of Medicine, provided the lists of employees and their workplace location (in cases of Schools with multiple teaching/research sites) and their e-mails. The invitation to participate in the survey was first sent by mail, asking the respondent to print the survey, fill it in and leave it in a special box at the main office of their institutions in 2012 to ensure full anonymity. No personal data were collected. The participation was voluntary and there were no incentives for participation; filling in the questionnaire was considered as the consent to participate. Three reminder e-mails were sent before and during the study period. Also, when the collectors came for the survey box, they also gave printed survey to university members who wanted to take part in the survey but did not print it from their e-mail earlier or had not received the e-mail. To preserve participant anonymity, sealed ballot-boxes were used for survey collection and the researchers collected the survey boxes at least 1 day after the surveys had been handed out. The survey collection boxes were opened only when the collection phase was complete.

Concurrently, early-stage researchers from the three schools were asked to participate in the survey of organizational climate and research integrity pressures. The eligible participants were doctoral and postdoctoral students (research fellows). The participation in the surveys was voluntary and anonymous. The same method of survey delivery and collection was used as for the ethics climate survey. As the survey for the junior researchers consisted of 113 questions, we offered a token for the completion of the surveys in the form of a €7 gift voucher from a local bookstore.

### Surveys

We used the 36-item *Ethical Climate Questionnaire* (ECQ) modified by Cullen et al. (1993). All of the items were graded on a 5-point Likert-type response scale, and detected one of nine possible climate types determined by the highest score of a 4-item group reflecting 3 levels of ethical analysis (individual, local, and cosmopolitan) and 3 levels of ethical criteria (egoism, benevolence, and principle). The 9 types of climate were: (1) self-interest, (2) company profit, (3) efficiency, (4) friendship, (5) team interest, (6) social responsibility, (7) personal morality, (8) company rules and procedures, and (9) laws and professional codes. Nine theoretical climate types had been empirically validated in previous research (reviewed in Simha and Cullen 2012). The English version of the ECQ had been translated into Croatian by the authors (AM and MMal) and then back translated by an independent language expert not involved in this study, in order to confirm the validity of the translated ECQ. The overall reliability of the ECQ in our sample was satisfactory (Cronbach’s α = 0.891).

The *Research Integrity and Organizational Climate* (RIOC) *survey* was developed by Gaddis et al. (2003), with the Croatian versions validated and further developed by Katavic et al. (2006). The survey consisted of four subscales, with items graded on a 5-point Likert-type response scale: (1) individual pressures, (2) group pressures, (3) organizational pressures, and (4) organizational climate. The reliability of the subscales was satisfactory (Cronbach’s α was 0.898, 0.931, 0.746, and 0.867, respectively).

### Statistical Analysis

Frequencies and percentages were used for the description of categorical variables. Differences between the sociodemographic characteristics of respondents were determined using χ^2^-test, and differences in total scale and subscale scores with Kruskal–Wallis test and post hoc Mann–Whitney *U* test. Scale scores are shown as means with 95% confidence intervals. The level of significance for all statistical tests was 0.05. Data were analysed with SPSS statistical package 19.0 (SPSS; Chicago, Illinois, USA).

## Results

The overall response rate from the University Schools’ faculty, administrative and other personnel in the ECQ survey was 49% (294 out of total 597 eligible participants): 131 (53%) respondents from the School of Electrical Engineering, Mechanical Engineering and Naval Architecture, 73 (46%) from the School of Humanities and Social Sciences, and 90 (47%) from the School of Medicine. The respondents from the three schools did not differ in the distribution of faculty/administration positions, but had discipline-based gender distributions that were characteristic for engineering (predominantly male) versus humanities/medicine (predominantly female) (Table 1). The respondents from the School of Humanities and Social Sciences had significantly shorter employment than those from the other two schools and respondents from the School of Medicine were the oldest (Table 1).

**Table 1:** Sociodemographic characteristics of faculty and staff at three different schools of the University of Split

Characteristics	No (%) of employees at the school of			*P*
	Engineering (n = 131)	Humanities (n = 73)	Medicine (n = 90)	
*Gender*				
Male	94 (72)	15 (21)	35 (39)	< 0.001*
Female	35 (27)	58 (79)	53 (59)	
Missing response	2 (1)	0	2 (2)	
*Age* (median, interquartile range)	38 (30–49)	38 (32–46)	49 (35–56)	< 0.001^†^
*Position*				
Faculty	89 (68)	49 (67)	62 (69)	0.950*
Staff	38 (29)	20 (27)	24 (27)	
Missing response	4 (3)	4 (6)	4 (4)	
*Years of employment* (median, interquartile range)	8 (3–20)	5 (4–7)	10 (5–19)	< 0.001^‡^

*χ^2^ test

^†^Medicine: *P* < 0.001 versus engineering, *P* < 0.001 versus humanities; Kruskal–Wallis test and post hoc Mann–Whitney *U* test

^‡^Humanities: *P* = 0.014 versus engineering, *P* < 0.001 versus medicine; Kruskal–Wallis test and post hoc Mann–Whitney *U* test

The overall response rate in the RIOC survey was 85% (70 out of a total of 82 eligible young researchers): 40 (87%) respondents from FEE, 13 (87%) from SHSS, and 17 (81%) from SM. This sample also showed a typical discipline-based gender distribution in engineering versus humanities/medicine (Table 2). There were no differences in their age, but young researchers at the School of Medicine were employed for a shorter time that at other two Schools (Table 2).

**Table 2:** Sociodemographic characteristics of young researchers working at three different schools of the University of Split

Characteristics	Young researchers at the school of			*P*
	Engineering (n = 40)	Humanities (n = 13)	Medicine (n = 17)	
*Gender (n, %)*				
Male	30 (75)	5 (38)	6 (35)	< 0.001*
Female	10 (25)	8 (82)	11 (65)	
*Age* (median, interquartile range)	29 (27–34)	33 (31–34)	28 (28–31)^‡^	0.11^†^
*Years of employment* (median, interquartile range)	4 (2–7)	4 (3–6)	3 (1–3)	0.020^‡^

*χ^2^ test

^†^Kruskal–Wallis test

^‡^Medicine: *P* = 0.012 versus engineering and *P* = 0.036 versus humanities, Kruskal–Wallis test and post hoc Mann–Whitney *U* test

### Ethical Climate

Overall, the dominant ethical climate type perceived at the three University Schools was *Laws and professional codes*, which is associated with the cosmopolitan level of analysis and the ethical construct of principle (Table 3). When analysed individually, this climate type was also predominant at both the Engineering and the Humanities Schools (Table 3). In contrast, the predominant ethical climate at the School of Medicine was *Self*-*interest*, which is associated with the individual level of analysis and the egoism ethical construct (Table 3).

**Table 3:** Perceived ethical climates at three School of the University of Split

Levels of ethical criteria	Levels of ethical analysis		
	Individual	Local	Cosmopolitan
*Overall*			
Egoism	12.2 (11.7–12.6) Self interest	10.1 (9.8–10.5) Organizational interest	11.2 (10.8–11.7) Efficiency
Benevolence	9.2 (8.8–9.7) Friendship	10.0 (9.5–10.5) Team interest	11.6 (11.2–12.0) Stakeholder orientation
Principle	11.4 (11.1–11.8) Personal morality	12.6 (12.1–13.0) Organizational rules and procedures	**12.8 (12.3–13.2)** **Laws and professional codes**
*School of Electrical Engineering, Mechanical Engineering and Naval Architecture*			
Egoism	11.9 (11.3–12.6) Self interest	10.0 (9.5–10.5) Organizational interest	11.0 (10.3–11.6) Efficiency
Benevolence	9.4 (8.8–10.1) Friendship	10.0 (9.3–10.7) Team interest	11.8 (11.2–12.4) Stakeholder orientation
Principle	11.3 (10.8–11.9) Personal morality	12.8 (12.1–13.4) Organizational rules and procedures	**13.0 (12.3–13.7)** **Laws and professional codes**
*School of Humanities and Social Sciences*			
Egoism	11.4 (10.5–12.3) Self interest	9.9 (9.2–10.6) Organizational interest	12.1 (11.2–13.1) Efficiency
Benevolence	9.9 (8.8–11.1) Friendship	10.8 (9.6–12.1) Team interest	11.8 (10.8–12.9) Stakeholder orientation
Principle	11.0 (10.3–11.8) Personal morality	13.0 (12.1–13.9) Organizational rules and procedures	**13.4 (12.4–14.4)** **Laws and professional codes**
*School of Medicine*			
Egoism	**13.1 (12.3–13.8)** **Self interest**	10.4 (9.9–11.0) Organizational interest	11.0 (10.2–11.7) Efficiency
Benevolence	8.5 (7.7–9.2 Friendship	9.4 (8.6–10.3) Team interest	11.1 (10.4–11.9) Stakeholder orientation
Principle	11.8 (11.2–12.4) Personal morality	11.9 (11.2–12.6) Organizational rules and procedures	12.0 (11.2–12.8) Laws and professional codes

Nine climate types are indicated in respective table cells. The results are expressed as a mean score out of the total of 20 items for each climate type (with 95% confidence intervals). The result for the dominant ethical climate type is emphasized in bold

The lowest overall score for all three university schools was the climate of *Friendship* (individual level of analysis and benevolence ethical construct); the score at the School of Medicine for this climate was the lowest among the three schools (Table 3).

Overall, years of employment positively correlated with the *Self*-*interest* climate type (r = 0.15, 95% CI 0.03–0.26; *P* = 0.013). *Self*-*interest* climate type was more often perceived as the organizational climate by the staff compared to faculty [mean (95% CI): 12 (11–12) vs. 13.5 (12–14), *P* = 0.005; Mann–Whitney *U* test]. There were no differences between these groups in the perception of the *Laws and professional codes* climate type [mean (95% CI): 14 (13–14) vs. 13 (12–14), *P* = 0.226; Mann–Whitney *U* test].

### Research Integrity and Organizational Climate

Early-stage researchers from the three University schools did not differ in their perceptions of organizational research integrity climate, or in their perceived individual, group or organizational pressures (Table 4).

**Table 4:** Organizational climate for research and situational influences as experienced by young researchers at three schools of the University of Split

Construct (score range)	Score (95% CI) of young researchers from the school of			*P* ^†^
	Engineering (n = 40)	Humanities (n = 13)	Medicine (n = 17)	
*Organizational climate inventory* (22–110)	72.0 (69.0–75.0)	75.5 (69.0–81.9)	71.6 (64.7–78.6)	0.195
*Situational influences (pressures) at level*				
Individual (41–205)	107.6 (101.4–113.9)	108.4 (98.4–118.4)	114.3 (103.8–124.8)	0.574
Group (32–160)	90.8 (85.1–96.4)	86.8 (74.8–98.7)	93.6 (82.6–104.7)	0.492
Organizational (17–85)	49.1 (46.5–51.7)	47.6 (43.3–51.9)	53.6 (49.3–57.9)	0.081

Constructs according to the instruments developed and validated by Gaddis et al. (2003)

^†^Kruskal–Wallis test

The analysis of subscales for each of the four constructs also demonstrated no differences among the perceived individual or group pressures (8 subscales for each; Gaddis et al. 2003) and in the overall organizational climate by young researchers from the three Schools. In the construct of Organizational pressure, statistically significant differences were found for two subscales, *Interdependence* and *Munificence*. The *Interdependence* score was highest for the researchers from the School of Engineering (mean score out of maximum 20 was 12, 95% CI 12–15, *P* = 0.015) versus researchers from either the School of Humanities (mean score = 11, 95% CI 10–12) or the School of Medicine (mean score = 11, 95% CI 10–12; *P* = 0.827 vs. Humanities). On the other hand, the *Munificence* score was significantly lower for the researchers from the School of Medicine (mean score out of maximum 15 was 10, 95% CI 8–11, *P* = 0.026) versus researchers from either the School of Engineering (mean score = 12, 95% CI 11–13) or the School of Humanities (mean score = 12, 95% CI 9–13).

There was a weak negative correlation between researchers’ years of service and *Expertise of the Major Professor* subscale in the Group pressure construct (rho = − 0.279, 95% CI − 0.04 to − 0.477, *P* = 0.013). There were no significant gender differences in the scores on individual constructs (data not shown).

## Discussion

There is little research on ethical climate at universities and research organizations (Acar et al. 2016; Acharya 2005; Al-Omari 2013) compared to the large body of research on ethical climate in business (Martin and Cullen 2006; Simha and Cullen 2012), and health care institutions (Koskenvuori et al. 2017). We studied the schools of engineering, which focuses on the technical aspects of research and professionalism, and the humanities and social sciences—where reflection and introspection into ethical issues is a part of the education process, and medicine—where professional deontology and medical ethics is formally a part of the curriculum. Overall, *Laws and Professional Codes* was the dominant perceived ethical climate type for the pooled results from all three schools. When analysed individually, *Self*-*Interest* emerged as the predominant ethical climate at the School of Medicine whereas *Laws and Professional Codes* remained the dominant ethical climate at the other two schools.

These two ethical climate types are among the most common empirical derivatives of the theoretical strata of ethical climate (Martin and Cullen 2006; Simha and Cullen 2012): (1) *Laws and Professional Codes* climate translates to *Law and Code* climate, in which ethical decisions are made based on codes external to the institutions, such as laws and professional codes of conduct; and (2) *Self*-*Interest* climate translates to *Instrumental* climate, in which the employees perceive their institution’s norms and expectations as encouraging for ethical decision making from an egotistic point of view and for behaviour promoting self-interest.

While this is, to the best of our knowledge, the first comparative assessment of the ethical climate at a university medical school, the results of our study should be interpreted with several limitations/caveats in mind apart from the general bias in using self-report surveys. Firstly, our results were obtained at a single university and a single medical school, so generalizations to other institutions are not possible. Secondly, the response rate was around 50%, which was similar to those reported for other studies at universities (Acar et al. 2016; Acharya 2005; Al-Omari 2013). We could not compare the characteristics of responders and non-responders in our study as the survey was completely anonymous and there is no available data on the demographic characteristics of the faculty at the university schools included in the study. Thirdly, there were differences in the overall mean age of the faculty and personnel at the three schools in our study, as well as the duration of employment, both of which were highest at the School of Medicine, which may have contributed to the observed differences among the schools.

We could not identify studies in the literature that used the ECQ in a medical school setting to compare our finding of a specific ethical climate in the medical school compared to other university schools. The tree studies that measured ethical climate in a university setting did not follow the classical theoretical strata or common empirical derivatives of ethical climate (Martin and Cullen 2006; Simha and Cullen 2012), so it was difficult to compare their results with those from our study. In the report of Al-Omari (2013) on ethical climate in a university in Jordan (without specification of the involved faculties), the predominant climate was identified as “egotistic”, without clarification of the individual, local or cosmopolitan locus of analysis. However, this type of climate would be similar to the common empirical *Instrumental* climate, which is associated with the egoism ethical construct and either individual or local locus of analysis (Martin and Cullen 2006; Simha and Cullen 2012), and thus similar to the climate observed at the School of Medicine in our study. In the study of the staff of Usak University in Turkey (again not specifying the university schools involved), Acar et al. (2016) reported the predominance of “Laws, rules and policies” ethical climate, similar to the overall result for the University of Split in our study. In the study of Acharya (2005), which involved a single dental university school in India, the reported (equally) predominant climates were those of “Consensual Morality”, “Self-Centered Morality” and “Universal Morality”.

Without underlying evidence, it is also difficult to explain the differences between the School of Medicine and the other two University Schools, one in the area of engineering and the other in humanities and social sciences. The overall predominance of *Laws and Professional Codes* ethical climate at the University of Split is not surprising for a country with a tradition of only public universities and strict national regulation of the research and education processes (Official Gazette 2003). While the universities have full academic and research freedoms, the funding, accreditation and legislative regulation of universities are at the national level, including the rules for institutional and individual academic/research assessment. This dependence on external codes and national regulation, and its reflection in the overall climate at the university, is supported by the findings from the survey measuring the research organizational climate from the perspective of early researchers according to the measures developed by Gaddis et al. (2003). In our study, we found no differences at the individual and group levels between the three Schools. Young researchers at all three schools, who had been predominantly employed through a national program of junior researchers, funded by the Ministry of Science, Education and Sports (Petrovečki et al. 2008), reported the experience of comparable levels of stress and competitive pressure (at the individual level) as well as comparable degrees of guidance by their superiors or group cohesiveness/conflicts (at the group level). These results suggest that young researchers, generally, did not have different perceptions of the levels of ethical climate within their institutions at the individual and group levels. These results should be interpreted keeping in mind the limitations of the measure instrument, which is not specific for each discipline and is contextualized only in a general way (Gaddis et al. 2003). We did not use the instrument to assess a single institution but rather to gain insight into the comparable experiences of young researchers across disciplines. The experiences of young researchers differed at the organizational level. Specifically, the score for the *Interdependence* construct was highest at the School of Engineering, and the score for the *Munificence* construct was lowest at the School of Medicine. The *Interdependence* construct measures the perception of how one’s work is influenced by the institution (e.g. how often the research had to be interrupted because the institution did not have enough resources; how often collaboration with other institutions was needed; how often researchers had to seek training outside of the institution to increase their expertise); the higher score on this construct means the smaller the contribution of the institutions to the research efforts of individual researchers and research groups. The *Munificence* construct measures the perception of how generous the institution is towards one’s research group (e.g. access to technical equipment and literature; work on several research grants at the same time, support for travel, conferences and professional meetings; researchers helping each other); a lower score indicates a smaller satisfaction with the support from the institution. Thus, one can argue that the experience of young researchers at the School of Engineering, compared to other schools, was that the forces beyond the immediate institutional reach hindered their work. In contrast, young researchers at the School of Medicine experienced a lack of fiduciary and other kinds of support by their own institution, which they felt was within the institutions’ reach. Both of these perceived lacks of support may negatively influence scientific productivity and the perception of overall levels fairness and ethics, thus having a chance of seriously influencing the scientific and ethical behaviour (Gaddis et al. 2003).

In the *Instrumental* climate, which was predominant at the School of Medicine, the employees perceive their institution’s norms and expectations as encouraging for ethical decision making from an egotistic point of view and for behaviour promoting self-interest (Martin and Cullen 2006; Simha and Cullen 2012). According to the large body of evidence, mostly in business organizations, as well as their meta-analytic syntheses, the *Instrumental* climate has the most negative influence on the consequences of ethical climate. It negatively influences the commitment of the individuals to the organization and their job satisfaction, while at the same time potentially promoting a wide array of undesirable behaviours, group under the common classification of “dysfunctional behaviour” in meta-analytic path analysis of the relationship between climate and individual level work outcomes (Martin and Cullen 2006). It is not clear whether this is also true for the climate we identified at the University school of medicine, and whether the findings from other disciplines, particularly business, could be directly applied to academic medicine. Medicine is a traditionally individual profession, with close relationships between doctors and patients as individuals, regardless of the current paradigm of team-work in medicine (Saba et al. 2012). The relationship between the doctor and the patient are from the position of power for a doctor and dependency for a patient (Kaba and Sooriakumaran 2007). This is also reflected in the WMA Declaration of Geneva modelled on the Hippocratic Oath (World Medical Association 2006), which doctors take at graduation from the medical school, and which puts the doctor and the patient in the centre of the profession. The Oath has been criticized for its lack of obligations to society (Cruess and Cruess 2014). Even in this critique of the individual-focus of the profession, doctors are considered to enter a social contract that grants them a privileged position in a society (Cruess and Cruess 2014). On the other hand, medical schools and their curricula may promote *Instrumental* ethical climates as described in other disciplines and types of organizations. The medical curriculum has been repeatedly shown to be a period for disillusionment for students, who start as young idealists (Branch et al. 1998) but get disillusioned by the hierarchical system in medicine (Hawkins 2003), which is later reflected in their professional work. Medical and other health professions students are also often confronted with ethical dilemmas for which they receive little support (Christakis and Feudtner 1993; Monrouxe et al. 2015), and the solutions for them are, often, to conform to the hierarchy and obey the norms and rules. Finally, students are exposed to a hidden medical curriculum (Hafferty and Franks 1994; Hren et al. 2011), which offers opposite values from the formal curriculum, which may lead students to turn inwards, leading to the perception that their studies are based on inconsistencies, contradictions and double messages. As the theoretical basis of the ethical climate constructs is related to Kohlberg’s theory of moral judgment (Rest et al. 2000), it is interesting that medical students often regress in their moral reasoning after they start clinical training during the medical curriculum (Hren et al. 2011). There is also evidence that different personality traits, as well as vocational interests influence not only work-related outcome, but also relationship- and health-related outcomes (Stoll et al. 2017), implying that individuals with certain traits may gravitate towards the climate that most reflects their personal climates. All of these factors, deeply embedded in the medical profession, may contribute to the ethical climate observed at the medical school in our study.

Our findings open many questions and point to future research into ethical climate perceptions in academic and research institutions. A large body of evidence from other types of organizations points to the powerful influence of ethical climate on organizational outcomes, both positive/desirable and negative/undesirable. Future questions for academic and research institution are several. Does ethical climate at a university medical school really differ from other schools? Are there cultural aspects of ethical climate, particularly in countries experiencing a transition from a controlled to a market economy, and burdened by corruption at all levels of society, including academia (Burazeri et al. 2005). Does ethical climate change over time and what are the drivers for change? Can ethical climate at an academic institution be managed for change? From a practical point of view, knowing the dominant ethical climates may enhance the understanding of the interpersonal and group dynamics at the work place, and could be used to tailor specific training programmes and continual education of faculty and administration personnel. We hope that our study will encourage more systematic and methodologically rigorous research into ethical climate in academic institutions so that we get greater knowledge and better tools to create the environment for academic and research integrity.
